# Experimental Researches Applied to Physiology and Pathology

**Published:** 1852-08

**Authors:** E. Brown-Séquard

**Affiliations:** Paris


MEDICAL EXAMINER,

AND

RECORD OF MEDICAL SCIENCE.

NEW SERIES.—NO. XCII.—AUGUST, 1852.

### ORIGINAL COMMUNICATIONS.

### *Experimental Researches applied to Physiology and Pathology.*

By E. BROWN-SÉQUARD, M. D., of Paris.*

#### I.—ON THE SOURCE OF THE VITAL PROPERTIES.

I think that every tissue possesses its vital properties, in consequence of its peculiar organization, and that in a completely developed animal, nutrition is the source of the vital properties, inasmuch as it is the cause of the maintenance of organization.

I will try to prove the correctness of my opinion, by the following remarks on some of the vital properties of the spinal cord, the nerves, and the muscles.

[* The paper which we have the pleasure of presenting to our readers from Dr. Brown Séquard, is a resumé of many researches made by the author, a part of which only have hitherto been published in any of the foreign journals.

The conclusions arrived at are the result of eight years exclusive devotion to the experiments upon which they are based.—EDS. EX.]

a.—*Source of the reflex faculty in the spinal cord.*

Notwithstanding the experiments of Redi, Whytt, Prochaska, Unzer, Sénac, Fontana, Caldani, Sir G. Blane, Fray, Legallois and many other experimenters; and notwithstanding the much more important experiments of Marshall Hall, Müller, Grainger, Volkmann, Kürschner, Pickford, de Martino, Buchner, Mayer, Paton and Stilling, the existence of the reflex faculty, after the spinal cord has been separated from the encephalon, is not considered by all physiologists as a proof of the independence of the spinal cord. J. W. Arnold and Flourens still maintain that the medulla oblongata is a centre, giving life to the other parts of the nervous system. The reflex faculty possessed by the spinal cord after it has been separated from the encephalon, is considered by J. W. Arnold as a remainder of something given to the spinal marrow by the encephalon, before their separation.

My experiments prove the incorrectness of that opinion.* I have found that after having exhausted the reflex faculty by putting it in action, energetically and frequently, in an animal on whom the spinal cord is separated from the encephalon, it reappears, and becomes soon as energetic as before, provided that the circulation of blood takes place in the cord. Moreover I have found, that if the reflex faculty is put in action frequently, it is able to produce an immense quantity of action: thus, for instance, it can stimulate sufficiently the muscles of a frog's leg to make them raise, in an hour and in divided portions, about twelve pounds, to the height of about two lines. In a pigeon the reflex faculty is able to stimulate the muscles of a leg so far as to make them raise fifty pounds, by fractions, in an hour, to the height of more than one inch.†

I shall add two other decisive proofs :—1. The reflex faculty is very weak in frogs immediately after the spinal cord has been separated from the medulla oblongata, and it increases afterwards, as R. Whytt and Marshall Hall have discovered. I have stated that it increases so much that the posterior limbs are able

* See :—Recherches et expériences sur la physiologie de la moelle épinière. Thèse inaugurale. Paris, 3 Janvier, 1846.—Comptes rendus des séances de l'Académie des Sciences. Paris, 1847 T. xxiv. p. 849.

† See Gaz. Méd. de Paris. T. 4. 1849. p. 233.

to draw up, by reflex action, more than double the weight the animal could raise up by an action of its will before the division of the cord. 2. After having divided the spinal cord in the dorsal region on a mammal, I kill it by cutting the right carotid artery. A few minutes after the cessation of reflex action I inject blood by the opening made in the carotid. Then life returns and with it the reflex faculty.

All these facts demonstrate positively that the reflex faculty is a vital property belonging to the spinal cord, and that its source is in the nutrition which maintains the organization of that nervous centre.

b.—*Source of the vital property of the motor nerves.*

The independence of the motor nerves is denied by almost all physiologists. They believe that the nervous centres are the sources of the vital property of these nerves. They base their opinion on this fact, that the motor nerves separated from the nervous centres soon lose their property, as it has been seen by Fontana, Haighton, Astley Cooper, Steinrueck, Müller, Sticker, Günther, Schoen, Kilian, Stannius, Helmholtz, Martin-Magron and others.

But, *in the first place*, if the motor nerves of the warm-blooded animals lose their vital property after having been separated from the nervous centres, it is not less positive that they retain it during several days. *Secondly*, if the vital property of the motor nerves is exhausted by very energetic action, it reappears after a short time, although the nerves are separated from the cerebro-rachidian centre, provided that the circulation of blood continues in them. *Thirdly*, if the circulation of blood is stopped in a limb in which the nerves have been divided, it is found that the peripheric portion of the divided nerves lose their vital property before the muscles. After the nerves have been left dead, *i. e.*, deprived of their vital property for a quarter of an hour, half an hour, and even more, blood is allowed to circulate anew in the limb. Then the vital property of the cut nerves returns, and, to produce a muscular contraction, only a slight compression upon them is necessary.* If the

* See Comptes rendus de l'Acad. des Sciences. T. xxxii. Séance du 9 Juin, 1851.—Gaz. Médic. de Paris. 1851. T. vi, p. 359.

motor nerves lose their property when they are separated from the nervous centres, it is because they are then badly nourished. Nerves as well as muscles must be exercised, in order to be well nourished.

c.—*Source of the muscular contractility.*

Although there are some facts which appear strongly to prove that the vital property of the muscular tissue is independent of the nervous system, many physiologists persist in their opposition to Haller's doctrine on this subject. Therefore I have thought necessary to add new proofs to those already known, and I have published many experiments, of which I shall relate here only two of the most decisive.*

1. The sciatic and the crural nerves having been resected, for ten or twelve days, on a rabbit or a guinea-pig, I examine if these nerves have completely lost their vital property, and if the muscles are still contractile. When this has been ascertained, I put a ligature around the aorta. Then muscular irritability disappears after a certain time and cadaveric rigidity appears. Three quarters of an hour or even an hour after the complete disappearance of the muscular irritability, and the appearance of the *rigor mortis*, I cut off the ligature, and I find, after ten or fifteen minutes, that the rigidity disappears and the contractility reappears. I need not say that the nerves do not regain their lost property. This fact clearly proves that the contractility is given to the muscles by blood, *i. e.*, by nutrition, and not by the nervous system.

2. Many experiments have shown to me that muscles paralyzed for five days or a little more, in consequence of the division of their nerves, remain much longer contractile after the death of the animal than the non-palsied muscles. This would hardly be the case if the contractility was given to muscles by the nervous system.

* See:—Bulletin de la Soc. Philomat. 1847, p. 74.—Gaz. Méd. de Paris, 1851, t. vi. p. 619, and 1852, t. vii. p. 72.

II.—RESEARCHES ON THE REFLEX FACULTY.

During the last seven years I have published many papers relating to the reflex faculty.* Among the facts which I have discovered I will mention the following:

1. Grainger had found that the act of suckling can be executed by an animal deprived of its brain. I have found that even after the ablation of both the brain and the cerebellum, newly-born rabbits are able to suckle very well; which is a proof that suckling may be executed by reflex action.

2. It is commonly affirmed that the reflex power is much stronger in cold-blooded than in warm-blooded animals. This opinion is correct so far as regards the contrast between Mammals and Batrachia (the animals usually compared); but it is incorrect if Birds are compared with Reptilia and Fishes. It has been said that the higher an animal is in the scale the less it has reflex power. If this be true, we should find more and more reflex power from Mammals to Fishes; but the real order, according to my experience is: 1st, Fishes; 2d, Mammals; 3d, Amphibia and Reptilia; 4th, Birds; so that Birds have more reflex power than all the other animals, and Mammals have more than Fishes. Of course, there are exceptions to this rule in the case of particular species; thus the eel, carp and tench have as much reflex power as many Mammals possess.

It has also been commonly affirmed that the reflex power diminishes with age, being the greatest in young animals. This statement, also, has been based on a too limited induction. In Reptiles and Fishes no difference can be detected in this particular. In Birds it is decidedly the other way, the reflex power being much the strongest in adults. Among Mammals the difference is usually in favor of the young animal; not, however, at the very earliest part of its life, but ten or twelve days after birth. As to man the reflex power appears to be greater in him than in Fishes and Mammals; but it is not so energetic as in Birds and in Amphibia.

I have found that the causes of the differences between differ-

* See my Inaugural Dissertation, Paris, 3 Janvier 1846, 1ere partie.—Comptes rendus de l'Acad. des Sciences, 1847, t. xxiv. pp. 363 et 859.—Gaz. Med. de Paris, 1849, t. iv. pp. 430 et 644; et 1850 t. v. pp. 98 et 476.

ent animals, as regards the energy of their reflex power, are to be explained by anatomical differences. There exists a constant relation between the degree of the reflex power and the amount of grey matter in the spinal cord. It appears, also, that the mode of circulation of the blood in the spinal marrow has a great share in the causes of differences amongst different animals.

3. It is not necessary for the existence of the reflex power that the spinal cord should be without alteration. I have found the reflex faculty remaining in pigeons after I had crushed the spinal cord, and produced in it a considerable alteration. This is important to be known by practitioners, to prevent their drawing the conclusion, from the existence of reflex action after a fracture or a luxation of the vertebral column in man, that the spinal cord is healthy.

4. The influence of the nervous system on the secretions, by a reflex action, has been very little studied. I will state two examples of these reflex secretions: 1st, There is on the face, and particularly on the forehead and the nose, an abundant production of sweat when the nerves of the taste are strongly excited, as they are, for instance, by common salt, pepper, sugar, etc. In certain persons the quantity of sweat produced in such cases is sometimes, even in the winter, very considerable. 2d, I have observed that it is sufficient to excite the nerves of taste in order to produce the secretion of gastric juice, bile and pancreatic juice.

### III.—RESEARCHES ON THE INFLUENCE OF THE NERVOUS SYSTEM UPON THE FUNCTIONS OF ORGANIC LIFE.

My experiments have convinced me that if it is certain that the nervous system is able to act, and frequently does act, on the functions of organic life, it is not the less certain that the action of the nervous system on these functions is not necessary. I hope this will be sufficiently demonstrated by the numerous facts I have to relate.

a. *Influence of the section of nerves on nutrition and secretion.*

1. The frequent occurrence of certain pathological changes after section of the sciatic nerve in Mammals, has been cited as a proof of the dependence of the nutritive operations upon nervous agency. I think the following experiments give evidence against that doctrine. I have divided the sciatic nerve in a number of rabbits and guinea-pigs, and placed some of them at liberty in a room with a paved floor, whilst I confined others in a box, the bottom of which was thickly covered with bran, hay and old clothes. In a fortnight, the former set exhibited an obviously disordered action in the paralysed limbs; the claws were entirely lost; the extremities of the feet were swollen, and the exposed tissues were red, engorged, and covered with fleshy granulations. At the end of a month, these alterations were more decided, and necrosis had supervened in the denuded bones. On the other hand in the animals confined in the boxes, no such injuries had accrued; and although some of them have been kept living for four, five and even six months after the division of the sciatic nerve, no alteration whatever has appeared in the palsied limbs except atrophy. In these cases a portion of the nerve had been cut off, so that reunion was nearly impossible and did not take place.

Experiments made on pigeons have given the same results.

It is obvious from these experiments that the pathological changes which occur after the section of the sciatic nerve do not proceed directly from the absence of nervous action, but that they are consequent upon the friction and continual compression to which the paralysed limbs are subject, against a hard soil, owing to the inability of the animal to feel or avoid it.

In similar experiments made on frogs, I found that no alteration took place, except when water penetrated through the wound, under the skin, and between the muscles.*

2. With the help of an eminent micrographer (Dr. Lebert), I have made researches on the influences produced on the capillary circulation in consequence of the section of all the nerves (sympathetic and cerebro-spinal nerves) in the legs of a number of frogs. We have found no appearance of trouble in the capillary circulation, neither in an hour, nor in three or four days after the division of the nerves.

3. When resection of a long portion of one of the sciatic and the crural nerves is made on a very young rabbit, guinea-

* See Gaz. Méd. de Paris, 1849, t. 4, p. 880.

pig or pigeon, the palsied limb continues to grow in length, but it grows only very little, if at all, in thickness. When the experiment is made on all the nerves of the wing in a very young pigeon, it is also found that the wing grows in length, but very little in breadth or in thickness. The secretion of quills takes place equally as well in the palsied limb as in the other.

The difference in all these cases between the length of the sound and that of the palsied limb or wing is never very considerable; nevertheless the length of the healthy parts is greater than that of the paralysed parts.

4. I have found that burns, wounds and ulcerations existing in parts palsied in consequence of the section of their cerebro-spinal nerves, are cured as quickly and as well as those in sound parts.

5. Atrophy is a constant consequence of the section of the nerves of a limb. I have found that it supervenes not only in the muscles and the bones, as J. Reid has discovered, but also in the skin, which becomes evidently thinner.

6. Krimer asserts that after the section of the nerves of a limb in Mammals, the venous blood is of a bright red color like the arterial blood. (Physiologische Untersuchungen, Leipzig, 1820, p. 138 exp. 1, and p. 152 exp. 9.)

Long before the publication of Krimer, Arnemann had declared that the blood appeared darker than usual in a limb on which all the nerves had been cut. (Versuche über die Regeration an lebenden thieren, Gottingen, 1786, t. i., p. 48.)

Longet (Traité de Physiologie, Paris, 1850, t. ii., B. p. 92,) says that he has seen the venous blood retaining its ordinary color even three days after the section of the nerves of the anterior limb in dogs.

Who is right—Krimer, Arnemann or Longet? Neither of them is perfectly right. The assertion of Arnemann is entirely incorrect. By experiments made on dogs, rabbits, guinea-pigs and pigeons, I have found that the venous blood in palsied limbs is evidently less black than it is in sound limbs. But it is not true to say that venous and arterial blood in paralysed limbs have the same color. It is always very easy to distinguish one from the other.

The transformation of the arterial blood into venous is not so complete in the palsied as in the sound limb, but it always takes place even in a great measure. There is a good proof of this in the result of my experiments on the hand and forearms of two decapitated men. I injected blood in the arteries of these parts thirteen or fourteen hours after death and when cadaveric rigidity existed. Surely there was in that case no nervous action whatever, and nevertheless the blood, which was of a bright red color when injected, came out nearly black from the veins!

From all these facts I shall conclude:

1st, That the nervous action (that of the sympathetic as well as that of the cerebro-spinal nerves) is not necessary for the change of color of the blood in the capillaries.

2d, That the nervous system of animal life has an influence upon nutrition by which it takes a share in the transformation of arterial into venous blood.

7. My friend Dr. Cl. Bernard has recently discovered the curious fact, that after the section of the sympathetic nerve in the neck, the face on the same side and more particularly the ear, become warmer and more sensible than the other side. The blood-vessels are much enlarged and a great many are visible which were not so before the operation.

I have found that the remarkable phenomena which follow the section of the cervical part of the sympathetic, are mere consequences of the paralysis and therefore of the dilatation of the bloodvessels. The blood finding a larger way than usual, arrives there in greater quantity; therefore the nutrition is more active. Now the sensibility is increased because the vital properties of the nerves are augmented when their nutrition is augmented. As to the elevation of the temperature, I have seen, as Dr. Bernard has, that the ear exhibits, sometimes, one or two degrees Fahr. more than the rectum; but it must be remarked that the temperature of the rectum is a little lower than that of the blood; and as the ear is full of blood, it is very easy to understand why it has the temperature of the blood. A great many facts prove that the degree of temperature and of sensibility of a part, is in close relation with the quantity of blood circulating in that part.

I base my opinion in part on the following experiments: If galvanism is applied to the superior portion of the sympathetic after it has been cut in the neck, the vessels of the face and of the ear after a certain time begin to contract; their contraction increases slowly, but at last it is evident that they resume their normal condition, if they are not even smaller. Then the temperature and the sensibility diminish in the face and the ear, and they become in the palsied side the same as in the sound side.

When the galvanic current ceases to act, the vessels begin to dilate again, and all the phenomena discovered by Dr. Bernard reappear.

I conclude, that the only direct effect of the section of the cervical part of the sympathetic, is the paralysis and consequently the dilatation of the bloodvessels. Another evident conclusion is, that the cervical sympathetic send motor nerve fibres to many of the bloodvessels of the head.*

8. Nearly all physiologists believe that the secretion of the gastric juice is stopped after the section of the two pneumogastric nerves. It is difficult to solve the question by experiments on warm-blooded animals, because they die too quickly after the section of the vagi. But it is not so with frogs. I have found that they are able to live perfectly well either after the extirpation of the medulla oblongata, or after the extirpation of the ganglia of the par vagum. In both these cases I have found that digestion continues to be performed. Consequently, if the gastric juice is necessary to digestion, it is certain that this liquid is secreted.†

* My experiments prove, also, that the bloodvessels are contractile, and that the nerves are able to put them in action. I have also to remark that it is a fact, well established by Budge and Waller, that the cervical sympathetic is one of the motor nerves of the iris, and that the spinal cord is the origin of the nerve-fibres going from the sympathetic to the iris. Some experiments, which I intend to perform again, appear to prove that the same parts of the spinal cord which give origin to some of the motor nerve-fibres of the iris, originate also the motor nerve-fibres going from the cervical sympathetic to the vessels of the head. Another conclusion is to be drawn from the results obtained by Budge, Waller, Bernard and myself; it is that the cervical sympathetic, instead of receiving its fibres from upwards to give them downwards, receives them downwards and distributes them upwards.

† Comptes rendus de l'Acad. des Sciences. Paris, 1847. T. xxiv. p. 363–64.

9. J. Reid has found, that if the four nerves uniting the spinal cord to the posterior limbs are cut across on both sides, in frogs, and if a galvanic current is applied every day to the palsied limbs on one side, these galvanized limbs retain their natural dimensions, while the palsied limbs not galvanised become atrophied.

I have found :—1. That if, instead of cutting only the four cerebro-spinal nerves of the posterior limbs, I divide also the branches of the sympathetic nerve which unite with them, the same results are obtained as in Reid's experiment. 2. That if a like experiment is performed on dogs, guinea-pigs, rabbits and pigeons, the same results are found. 3. That if after atrophy has taken place in the limb of a mammal or a pigeon, a galvanic current is applied, every day, during several weeks, the atrophy diminishes little by little and the limb at length becomes as large as a sound limb. This happens although there is no return of vital property in the divided nerves. 4. That if the application of galvanism is made on the palsied limbs of very young animals, and continued every day until they have arrived at adult age, these limbs are then found to have grown as much, in every respect, as the sound limbs.

In addition to these facts I have to state that in cases of lead palsy, in which the extensor muscles, as far as I have been able to judge, were completely destroyed and replaced by fibrous tissue, I have seen muscles created by galvanism and becoming as strong as they are in healthy men.

In a case, which I published two years ago, (Gaz. Méd. de Paris, 1850, t. v. p. 169,) I have found that an increase of five centimetres in circumference took place in the superior part of the leg of a young gentleman, under the influence of galvanism, applied three quarters of an hour each day for six days.

In all the facts before related, galvanism acts by two ways: the one is that it exercises the muscles, and increase in consequence their nutrition; the other is that it produces directly some of the chemical changes which constitute nutrition.

The atrophy, which happens in paralyzed muscles, takes place mostly because they remain without exercise, and partly because when nervous action is deficient the respiration of the muscles is not carried on as well as when the nervous system acts upon them. Galvanism applied to a palsied limb acts partly in producing the transformation of arterial into venous blood, *i. e.*, what Gustav Liebig calls the respiration of the muscles. I have seen frequently the venous blood, in palsied limbs, becoming as black as normal venous blood, after the application of galvanism. This change of coloration is not produced by a direct chemical influence, exerted by galvanism on the blood, for if galvanism is applied to blood in a vase, nothing of that kind is seen. It is in consequence of an interchange between blood and the living tissues that the change of color happens. The muscular contraction which takes place under the influence of the nervous system, or that of galvanism, produces, in both cases, an increase in the darkness of the venous blood. This fact proves that the consumption of oxygen by muscles is increased during their contraction.

I conclude from the preceding facts :—

1st. Nervous action is not necessary for nutrition.

2d. Atrophy in palsied limbs is more a consequence of absence of exercise than of any other cause.

3d. Muscular atrophy, at any stage, may be cured by galvanism.

b.—*Influence of the nervous centres on nutrition and secretion.*

1. Every one knows the singular alterations which take place in the eye after a contusion of the frontal nerve, or a section of the trigeminal or the cervical sympathetic nerves. Every one knows also that the existence of worms in the intestinal canal, and also certain affections of the spinal cord, are able to produce morbid phenomena in vision, and even diseases of the eye, and especially amaurosis. I have found that after the section of a lateral half of the spinal cord, it sometimes happens that the eye, on the same side where the cord has been wounded, will present strange and various alterations. The part of the cord having that influence on the eye, lies between the ninth and the twelfth costal vertebræ. The alteration exists generally in the cornea. In one case a ridge appeared on the anterior surface of that membrane four days after the operation. On the fifth day the ridge was deeper, and its edges had become opaque ; on the sixth day all the cornea was opaque. It remained so for five days, after which the opacity disappeared and no trace remained of it, or of the ridge. This experiment has been made on guinea-pigs.

2. I have found a considerable hypertrophy of the two supra-renal capsules, on eight or ten guinea-pigs, upon which a lateral half of the spinal cord had been cut in the dorsal region, for eight, ten or fifteen months. These organs had acquired, in some of these cases, three times their natural dimensions, and in others only the double. There was no appearance of change in their structure.

By an examination of the supra-renal capsules in guinea-pigs, on which I had made the section of a lateral half of the spinal cord, a few hours or a few days previously, I have found these organs congested, and sometimes containing even a slight effusion of blood. It is very probable that such a congestion has been the cause of the hypertrophy found in animals operated on at a much longer time previously. The congestion is certainly the result of a peculiar disturbance in the nervous action. A part only of the spinal cord appears to possess that singular influence on the supra-renal capsules. That part is extended, in guinea-pigs, from the tenth costal vertebra to the third lumbar. A simple puncture of the cord is frequently sufficient to produce the congestion of both supra-renal capsules.

3. The researches, made before mine, as to the influence of the spinal cord on the urinary secretion, could not give a decided result, because no physiologist had been able to keep any warm-blooded animal living a sufficient time, after the destruction of a large part of the spinal cord.

The results obtained by Ségalas on some animals who have lived from fifteen minutes to an hour after the destruction of the lumbar part of the cord, had led him to conclude that the spinal cord has no influence on the urinary secretion. Longet (Traité de Physiologie, Paris, 1850, t. ii. B. p. 199) says:—"Many observations have demonstrated to me that the visceral organs, which receive their nerves from the sympathetic, *are far from being immediately paralyzed by the section of these nerves, and that their action is even maintained much longer than the duration of the experiments in which Ségalas had destroyed the spinal*

*marrow.** Therefore I think I am allowed to maintain that after such an injury, the nerves going to these organs, and more particularly to the kidneys, do nothing but spend little by little the *nervous force*, originally and principally derived from the spinal marrow, which is the chief, if not the exclusive centre of its production ; thence the persistence of the renal secretion, as well as that of the movements of the heart, the intestinal canal, the uterine cornua, etc."

I could relate a great many experiments proving the incorrectness of Longet's theory, but a single one is sufficient. I have kept living, nearly *three months*, a young cat, on which the spinal cord had been *completely destroyed* from the eleventh or twelfth costal vertebra to its termination. This cat has lived all that time in apparently good health, and its *urine* has always been perfectly normal. It was acid, as is the case constantly in cats fed on meat, milk and bread. The bladder was palsied, but the sphincter vesicæ was generally contracted, so that every day I had to compress the abdomen and the bladder to empty this pouch. When I remained two days without doing that operation, the bladder contracted in consequence of the excitation produced on its muscular fibres by their distension.

This fact clearly proves that the urinary secretion is not under the dependence of the spinal cord.

According to Krimer, the medulla oblongata is the nervous centre upon which the urinary secretion depends. My experiments prove that this opinion is incorrect:—1st. After the destruction of the medulla oblongata in frogs, I have found that the secretion of urine remains as long as the animals have lived, *i. e.*, three or four months. 2d. On hybernating mammals, in winter time I have extirpated the medulla oblongata, after having emptied the bladder. These animals have lived a little more than a day, when I took the precaution of insufflating air in their lungs many times each hour. After their death the bladder was found full of urine apparently normal.

The medulla oblongata is not therefore a centre on which the urinary secretion depends.

4. The well known opinions of Segalas, W. Philip, Krimer,

* The italics are Longet's.

Chossat, Longet and others, about the influence of the spinal cord on the functions of organic life, are quite erroneous. I have found that birds are able to live for months after the destruction of the spinal cord, from the fifth costal vertebra to its termination. This fact proves not only that the functions of organic life may continue to exist in such a case, but that they appear to be executed then as in healthy birds; for, if the operation has been made on a young bird, it will afterwards grow very well.

I have succeeded in keeping alive, from the 8th of April until the 4th of July, a young cat, about which I have already published a note in this journal.* The palsied parts in this animal have grown in length proportionately as much as the sound parts. The growth has been such in the palsied limbs that they have acquired more than double the length they had at the time of the operation. The functions of organic life appeared to exist without any apparent disturbance. The nutritive reparation was so powerful, that the pieces of the vertebral column which had been cut off have been reproduced. This fact is important, because it shows that the reproduction of bone is possible in a palsied part.

The temperature of that cat was at the ordinary degree, (105° Fahr., in the rectum.) The secretion of the hair and nails took place as in healthy cats. I had previously seen on birds that their temperature remained normal after the destruction of a great part of the spinal cord. Besides, I have found in these birds that the secretion of quills and nails continued to take place.

As to the influence of the medulla oblongata on the functions of organic life, my experiments on cold-blooded vertebrata have proved to me, that these functions (except, of course, pulmonary respiration,) may continue to exist without any appearance of disturbance.

5. After the complete transverse section of the spinal cord in mammals or birds, I have found that the ulcerations which take place around the genital organs do not result directly from the absence of nervous action. One of the causes of these ulcerations is continued pressure, and another cause is the continual presence of altered urine and fæces.

* See Med. Exam., No. v. May, 1852, p. 321.

My opinion is well proved by the following experiments:—

1st. I have put, three or four times a day and for many days, a certain quantity of urine on the posterior part of the neck, in the neighborhood of the scapulæ, upon guinea-pigs. Before a week elapsed, the skin, at the place acted on by the urine, had lost its hair and epidermis. After a week more there was an ulceration in the skin, and ten or twelve days later the skin was destroyed, and there was an ulcer with a very bad aspect. This fact proves how powerful is the action of urine on the skin.

2d. On guinea-pigs, upon which the spinal cord was cut in the dorsal region, and on pigeons, upon which the spinal cord was destroyed from the fifth costal vertebra to its termination, I have found that no ulceration appeared when I took care to prevent any part of their bodies from being in a continued state of compression, and of washing them many times a day to remove the urine and fæces.

3d. In cases where an ulceration had been produced, I have succeeded in curing it by washing and preventing compression.

4th. I have found that in animals having the spinal cord cut across, every kind of wounds or burns were cured as quickly as in healthy animals.

Therefore the ulcerations which appear, in cases of paraplegia, do not exist directly in consequence of the palsy; they can be avoided and in many cases they can be cured.

These conclusions are perfectly true in animals having had an injury to the spinal cord for a shorter time than a year; but on guinea-pigs, upon which a lateral half of the spinal cord, had been cut for fourteen, fifteen, or eighteen months, near the tenth or eleventh costal vertebra, I have found an alteration of nutrition in the palsied parts. It was the right half of the spinal cord which had been cut, and in such a case, as I have discovered, the left side of the body behind the wounded part evidently loses a portion of its sensibility, and its temperature is also diminished. I have found, at the time designated, an ulceration coming in the part between the sacrum and the hip-joint. That ulceration has taken a tolerably great extension in surface but not in depth. It became as large as a half dollar. The part ulcerated has never been subjected to any kind of compression, neither to the action of urine and fæces.

Another kind of disturbance of nutrition occurred in these animals: they lost the hair of the leg, and of the other parts in which the sensibility was diminished.

6. It is known that erection is a frequent phenomenon in men after a fracture or a luxation of the vertebral column. It is known also, that in men hanged, erection and even ejaculation are not uncommon. Ségalas says he has seen these phenomena produced by the excitation of the spinal cord. Longet (loco cit., p. 201,) declares that he has not seen the excitation of the cord producing such effects. It is very easy to ascertain, on male guinea-pigs, that Ségalas is right.

1st. A transverse section of the spinal cord always produces an erection and an ejaculation.

2d. When one of these animals is asphyxiated, erection and ejaculation take place.

3d. If the spinal cord is galvanized, erection and ejaculation are produced.

These facts prove the influence of the spinal marrow on the seminal vesicles. They empty themselves slowly when the cord is galvanized.

In asphyxia, there are universal convulsions even in the muscles of organic life, as uterus, intestine, etc. These last organs are then put in contraction, and it is not astonishing, consequently, that the seminal vesicles become also contracted. The cause of these general contractions is the excitation of the spinal cord by venous blood, and very probably by a large amount of carbonic acid, as I will elsewhere try to demonstrate.

IV.—ON THE REPARATIVE POWER OF THE NERVOUS SYSTEM.

I have recently published in this journal* the results of my researches on the reparative power of the spinal cord. From these researches I have drawn the following conclusions:—

1st. That the spinal marrow, even in adult mammalia, may be exposed to the action of the air without danger to the life of the animal.

2d. That wounds of the spinal marrow may be repaired.

* Med. Exam., No. vi., June, 1852, p. 379.

3d. That after a complete transverse section of the spinal cord, the functions of that organ may be entirely restored.

As to the nerves, the experiments of Fontana, Haighton, Tiedemann, Flourens, Steinrueck, and many others, have demonstrated the possibility of reunion of the two extremities of a cut nerve. But, in most, if not in all these experiments, the return of sensibility and of voluntary movements have not been complete. The following fact is, consequently, very important, because it proves the possibility of a complete reappearance of the lost faculties after the entire division of a nerve.*

A guinea-pig, on which the sciatic nerve had been cut across, exhibited indications of a return of sensibility a month after the operation. Two months afterwards the sensibility was increased, but was still much inferior to that of the sound limb. The muscles then were beginning to contract under the influence of the will. Six months after the section, the animal could evidently move its legs and toes voluntarily; the sensibility then was almost entirely recovered. At the end of about eleven months, the sensibility and all the voluntary movements were apparently alike in the two posterior limbs. The animal having been killed, it was found by my friend Dr. Lebert and myself that, except a slight union of muscular fibres with the nerve at the place where it had been divided, the restoration of the original condition was so complete that no indication of the division could be discovered, either with the naked eye or with the microscope. I had seen the usual swelling of the nerve at the point of reunion about the sixth month after the operation, but at the time of the last examination it had disappeared.

### V.—ON TURNING AND ROLLING AS PHENOMENA PRODUCED BY INJURIES OF THE NERVOUS SYSTEM.

Pourfour du Petit and Méhée de la Touche were the first experimenters who witnessed turning produced by an injury of the nervous centres. But the first valuable researches on this phenomenon were made by Magendie and Flourens.

The parts of the cerebro-spinal centre which can be injured without producing turning, are: the cerebral hemispheres, the

* See Gaz. Med. de Paris, 1849, t. iv. p. 880.

cerebellum, the corpora striata, the corpus callosum, the spinal marrow and the olfactive and optic nerves.* All the other parts of the cerebro-spinal centres are able to produce turning or rolling.

These circulatory or rotatory movements take place sometimes on the same side, and sometimes on the side of the body opposite to that of the encephalon which has been injured.

A puncture of one of the following parts produces turning or rolling on the injured side:

1st. The anterior extremity of the thalami optici, according to Schiff.

2d. The crura cerebri, according to Magendie.

3d. The bi, or quadri-geminal tubercles, according to Flourens.

4th. The pons varolii.

5th. The posterior part of the processus cerebelli ad pontem.

6th. The auditive nerve, according to my own experiments.

7th. The medulla oblongata at the point of insertion of the facial nerve, according to my experiments in common with Dr. Martin-Magron.

8th. The medulla oblongata outside of the anterior pyramids, according to Magendie.

9th. A great part of the posterior face of the medulla oblongata, according to my experiments.

The parts of the encephalon which produce turning or rolling on the opposite side, are:

1st. The posterior extremity of the thalami optici, according to Schiff.

2d. The crura cerebri, according to Lafargue.

3d. The anterior part of the processus cerebelli ad pontem.

4th. A small part of the medulla oblongata before the nib of the calamus scriptorius and behind the corpora olivaria, according to my experiments in common with Dr. Martin-Magron.

Some of these two series of parts ordinarily produce turning and the others rolling. But these two kinds of movements can be produced by the puncture of a single part of the encephalon. Rolling is nothing but the exaggeration of turning; thus, after

* I consider as a part of the nervous centres, the three nerves of the superior senses: the olfactive, the optic and the auditive.

a puncture of the medulla oblongata, the animal at first rolls, and after some instants, instead of rolling, it turns. If, when it is turning, a slight puncture is made anew, close to the first, then the animal rolls.

Before trying to explain turning, I will give an outline of some of its species.

1st. *Turning and Rolling caused by tearing the facial nerve.*

My friend Dr. Martin-Magron and myself have discovered that if the facial nerve of a rabbit or a guinea-pig be exposed at its exit from the stylo-mastoid foramen, and be then drawn away from the cranium, so as to tear it asunder near its origin, the animal begins in about five minutes to turn itself round and round, the movement being from left to right when the nerve has been thus torn on the left side, and from right to left when it has been torn on the right side. This rotation is generally preceded by convulsive movements of the eyes, of the jaws, and of the head upon the trunk: and the body is then bent (as in pleurosthotonos) towards the injured side, by the contraction of all the longitudinal muscles of that side, the power of which is such as to resist considerable force applied to extend them. The movement at first takes place in a small circle; but the circle generally enlarges more and more, until at last, after twenty or thirty minutes, the animal walks in a straight line. There is no paralysis of any muscles, save the facial. The effect is not produced, unless the nerve be torn close to its origin.

When the nerve on the other side also is torn, even after a long interval, instead of the tendency to turn to one side, there is, at first, a rolling of the body on its longitudinal axis, which takes place towards the side last operated on. After this has continued, however, for twenty minutes or more, the animal recovers its feet, and begins to *turn*, as after the first operation, but towards the other side. This movement soon ceases.

Dr. Martin-Magron and myself think that the cause of these phenomena does not exist in the facial nerve itself, but in the part of the medulla oblongata from which this nerve has originated.*

* See Gaz. Méd. de Paris, 1849, t. 4, p. 879.

2d. *Turning and Rolling produced by an injury to the Medulla Oblongata.*

M. Magendie (Précis Elém. de Physiol. Paris, 1836, t. 1, p. 414) says: "Having raised up the cerebellum, I make a section perpendicularly to the surface of the fourth ventricle and at three or four millimetres from the median line. If I cut on the right, the animal will turn on the right side; if I cut on the left, it will turn on the left side."

If we suppose a plane cutting the medulla oblongata transversely at the distance of nearly two lines before the nib of the calamus scriptorius, the posterior face of the medulla oblongata will be divided into two parts: one before that plane, which I will call superior, and the other behind, or inferior.

Now, every puncture on that superior part produces turning or rolling on the side which has been punctured. The slightest puncture on the processus cerebelli ad medullam oblongatam is able to produce a violent and very rapid rolling. As long as the animal lives after the operation, it rolls or it turns at each time it tries to walk.

When (as Dr. Martin-Magron and myself have discovered) a deep section is made on the inferior part of the posterior face of the medulla oblongata, before the nib of the calamus scriptorius, turning is produced on the side of the body opposite to the punctured side of the medulla. A rabbit, which has lived thirteen days after the operation, had still the circulatory movement a few hours before dying. Nevertheless, sometimes the animal could walk nearly straight during a few seconds.

3d. *Turning Produced by a Puncture or a Section of the Acoustic Nerve.*

Flourens has discovered that, after the section of the semicircular canals, turning sometimes takes place.

I have found all the facts he relates about this subject perfectly right. It was interesting to know if a puncture or the section of the auditive nerve would produce turning. As it was impossible to operate on that nerve in mammals, I have experimented on frogs. In these amphibia it is easy to find the nerve and to act upon it. I have found that after a puncture or a section on the trunk of the nerve, the animal begins instantly to turn. As long as the frogs live, after a puncture of the acoustic nerve, they turn; but the circle of turning is much smaller a short time after the operation than afterwards. I have kept such frogs for months.

4th. *On a New Mode of Turning.*

I have found a mode of turning which has altogether some of the characters of turning and of rolling.

In the circulatory movement called turning (*mouvement de manége*), the body of the animal is bent on one of the lateral sides. It has the shape of an arch, and this arch is generally a part of the circumference described by the animal when turning. The smaller the radius of that arch, the smaller is the circle of turning.

In the new mode of turning I have found, the body of the animal is not bent, and when it walks it moves laterally, instead of going forwards. In turning it describes a circle, but the longitudinal axis of its body, instead of being then a part of the circumference, is a part of a radius, so that its head is at the circumference, and its tail towards the centre of the described circle.

That mode of turning has been executed by animals on which the quadrigeminal tubercles and the pons varolii, on one side, had been punctured by a pin. One of the eyes was convulsed; the other was in its normal condition. The convulsed eye was the right one, and the tubercles punctured were those of the left side.

5th. *On the Causes of Turning and Rolling.*

I have not room enough to show that the theories of Magendie, Flourens, Henle, Lafargue and Schiff are contradicted by a great many facts. I will only present the following remarks:

1st. As the slightest puncture of certain parts of the encephalon is sufficient to produce turning or rolling, it is evident that those rotating movements do not exist in consequence of an hemiplegia, as Lafargue, Longet and Schiff believe they do. Another reason is that every degree of hemiplegia exist in man without being accompained by turning or rolling. Besides, these phenomena have been observed in persons who had no paralysis at all.

2d. The theories of Magendie and Flourens are also opposed, by the fact that a slight puncture is sufficient to produce turning or rolling.

3d. As to the theory of Henle, which is based upon the existence of convulsions in the eye, producing a kind of vertigo, it has against it the facts that, on one side, convulsions may exist in the eyes without any other disorder in the movements; and, on the other side, sometimes turning or rolling exist without any convulsion in the eyes.*

Nevertheless, I think that, in many cases, the vertigo consequent on convulsions of the eyes is one element of the cause of turning. I think also that, in certain cases, paralysis of some parts of the body may facilitate the rotatory movements. But their great cause, I think, is the existence of a convulsive contraction in some of the muscles, on one side of the body. These convulsive contractions are to be found in every case of circulatory or rotatory movement. As to the cause of these contractions, it exists in the irritation produced in certain parts of the encephalon.

### VI.—ON A MEANS OF MEASURING DEGREES OF ANÆSTHESIA AND HYPERÆSTHESIA.

The curious facts discovered by E. H. Weber, on tactile sensibility, are well known. He found that if the two blunted points of a pair of compasses are applied simultaneously on the skin, there is, according to certain circumstances, either the sensation of one or of two points. When the points are both inside of certain boundaries, they are felt as one only; when they are outside of these boundaries, both are felt. These boundaries vary exceedingly in different parts of the skin, but for a given part the differences between men are not extremely considerable. I have made use of the compasses for measuring the degrees of tactile sensibility in diseases. In a case of considerable anæsthesia of the lower extremities, the patient only felt a single impression on one leg, although the

* See a very remarkable case observed by my friend Dr. Lebret, in *Comptes rendus* et Memoires de la *Soc. de Biologie* anneé 1850. Paris, 1851. t. ii. p. 7.

points of the compasses were ten, fifteen, or even twenty centimetres apart; whilst on the other leg he could distinguish them at a distance of twelve centimetres. The normal limit is generally, for that limb, from three to five centimetres. In another case where anæsthesia was slighter, the limit of the discriminating power was at from nine to sixteen centimetres. In two other cases, in which the diminution of sensibility had not been found by the other means of diagnosis, the compass indicated a very slight and beginning anæsthesia. The limit was at from six to seven centimetres.

These facts, and many others, have demonstrated to me that by the help of the compass, a physician can ascertain : 1st. Whether there is a slight anæsthesia or no. 2d. What is the degree of anæsthesia. 3d. What changes occur every day in the amount of anæsthesia.

The same is true for the cases of hyperæsthesia. In a case of paraplegia of the motor power, the patient felt the two points of the compasses, on his feet, even at the distance of five millimetres, whilst a healthy person feels the two points only when they are at a greater distance than twenty-five millimetres.

I shall add, that for succeeding in such experiments the two points must be blunted and applied simultaneously.*

(To be continued.)

* See Gaz. Med. de Paris, 1849, t. iv. p. 1012.

